# Characterization of *ftsZ* Mutations that Render *Bacillus subtilis* Resistant to MinC

**DOI:** 10.1371/journal.pone.0012048

**Published:** 2010-08-11

**Authors:** Inês Filipa Fernandes de Oliveira, Anabela de Sousa Borges, Viola Kooij, Jeremy Bartosiak-Jentys, Joen Luirink, Dirk-Jan Scheffers

**Affiliations:** 1 Bacterial Membrane Proteomics Laboratory, Instituto de Tecnologia Química e Biológica, Universidade Nova de Lisboa, Oeiras, Portugal; 2 Department of Molecular Microbiology, Vrije Universiteit Amsterdam, Amsterdam, The Netherlands; 3 Sir William Dunn School of Pathology, University of Oxford, Oxford, United Kingdom; Charité-Universitätsmedizin Berlin, Germany

## Abstract

**Background:**

Cell division in *Bacillus subtilis* occurs precisely at midcell. Positional control of cell division is exerted by two mechanisms: nucleoid occlusion, through Noc, which prevents division through nucleoids, and the Min system, where the combined action of the MinC, D and J proteins prevents formation of the FtsZ ring at cell poles or recently completed division sites.

**Methodology/Principal Findings:**

We used a genetic screen to identify mutations in *ftsZ* that confer resistance to the lethal overexpression of the MinC/MinD division inhibitor. The FtsZ mutants were purified and found to polymerize to a similar or lesser extent as wild type FtsZ, and all mutants displayed reduced GTP hydrolysis activity indicative of a reduced polymerization turnover. We found that even though the mutations conferred *in vivo* resistance to MinC/D, the purified FtsZ mutants did not display strong resistance to MinC *in vitro*.

**Conclusions/Significance:**

Our results show that in *B. subtilis*, overproduction of MinC can be countered by mutations that alter FtsZ polymerization dynamics. Even though it would be very likely that the FtsZ mutants found depend on other Z-ring stabilizing proteins such as ZapA, FtsA or SepF, we found this not to be the case. This indicates that the cell division process in *B. subtilis* is extremely robust.

## Introduction

Rod shaped bacteria divide precisely in the middle by forming a septum to produce two daughter cells. Formation of the division septum starts with the formation of the Z-ring, by self-assembly of the bacterial tubulin homologue FtsZ into a ring-like structure at midcell [1]. Z-ring formation is subject to tight control to ensure that cell division takes place at the right place and the right time. Placement of the Z-ring is controlled by two key systems that prevent formation of the FtsZ ring at the cell poles, or across the nucleoid: the Min system and nucleoid occlusion [2]. Nucleoid occlusion is mediated by the DNA binding proteins Noc in *Bacillus subtilis* [3] and SlmA in *Escherichia coli* [4]. These proteins become essential in bacteria in which the Min system is knocked out, but as yet their mode of action is unknown [3], [4]. The Min system, which was originally discovered in *E. coli* [5], prevents division at the cell poles. In the absence of *min*, polar division gives rise to spherical minicells that lack chromosomal DNA and elongated cells that contain two or more nucleoids.

The Min system is highly conserved and consists of two proteins that together act to prevent division at the cell poles [2], [6]. MinC and MinD form a dimer of dimers that binds to the cytoplasmic membrane. For *E. coli*, extensive studies have shown that membrane binding is mediated by MinD, which polymerizes and binds to the membrane in an ATP regulated fashion [6]. MinC forms the actual inhibitor of FtsZ polymerization [7]. Topological specificity to the MinCD inhibitor is conferred in different ways in Gram-positive and Gram-negative bacteria [2]. In Gram-positive bacteria, like *B. subtilis*, topological specificity is conferred by MinJ, a protein that localises to the cell poles in a DivIVA dependent manner and that forms a bridge between MinD and DivIVA to keep MinCD anchored to the poles [8]–[11]. In Gram-negative bacteria, like *E. coli*, the MinE protein imposes a pole-to-pole oscillation on MinCD by stimulating MinD ATPase activity which results in membrane detachment of MinCD [12]–[16]. The net result of both topology systems is that the concentration of MinCD is lowest at midcell, dropping below a threshold that allows Z-ring formation once the cell has reached a critical length [e.g. [17],[18]]. Recently, using MinC-GFP in *B. subtilis* at wild type expression levels, Gregory *et al.* showed that MinC relocalizes to the division septum shortly after the start of septum formation. Thus, MinC functions in preventing immediate initiation of another round of division at the newly formed cell pole [19].

MinC is the actual inhibitor of FtsZ and functions as a dimer [7]. Recently we showed that, like the *E. coli* MinC, *B. subtilis* MinC is an inhibitor of FtsZ polymerization, preventing lateral interactions between FtsZ protofilaments [20], [21]. We established that *B. subtilis* MinC activity is pH dependent, but that even at optimum pH (7.5) the inhibition of FtsZ polymerization is not as strong as originally described for *E. coli* MinC [7], [21]. MinC consists of two domains. The N-terminal Z-domain interacts with FtsZ and is a potent inhibitor of FtsZ polymerization and the C-terminal D-domain is required for MinD binding; both domains are involved in MinC self-association [22]. However, the C-terminal domain (MinCc) on its own also has some effect on FtsZ bundling [20], and when overproduced together with MinD, is capable of blocking division in *E. coli* [23].

Despite the fact that crystal structures of both MinC [24] and FtsZ [25] exist, not much is known about the interaction between FtsZ and MinC. Mutations in *E. coli* FtsZ that confer resistance to full-length MinC have all been identified indirectly, as mutations that principally conferred resistance to the *E. coli* FtsZ inhibitor SulA [26], [27]. SulA is expressed as part of the SOS response to DNA damage and binds directly to the FtsZ T7 loop which is a critical part of the FtsZ-FtsZ interface in a FtsZ polymer [28]. Notably, the lesions in five out of six mutants identified lie in the N-terminal GTP binding domain of FtsZ and probably affect the conformation of the GTP-binding pocket [27]. This would preclude SulA binding, but also affect the dynamics of GTP hydrolysis and polymerization of the mutants proteins, as has been shown for FtsZ2, which formed polymers with increased stability [29]. The MinC insensitive phenotype of these mutants could therefore be an indirect result of the increased polymer stability of these mutants. One mutation, *ftsZ103* (Phe268→Cys) mapped in the poorly characterized FtsZ C-terminal domain [26], but is localized between S8 and H10 in the FtsZ structure, which is covered by SulA when bound to FtsZ [28]. It is unlikely that SulA and MinC share a common binding site on FtsZ as SulA binding completely blocks both polymerization and GTP hydrolysis, whereas MinC blocks polymerization but has no effect on GTP hydrolysis [7]. In two recent studies, Shen and Lutkenhaus used the block in cell division caused by overproduction of the N- or C-terminal domains of *E. coli* MinC to isolate FtsZ mutants that have lost the capacity to interact with MinC [30], [31]. Mutations that render FtsZ resistant to the C-terminal domain of MinC mapped in the conserved C-terminal tail of FtsZ which is also the interaction site for ZipA and FtsA in *E. coli* [32] and EzrA and SepF in *B. subtilis* [33], [34]. Although mutations in the FtsZ C-terminus prevented the interaction of FtsZ with MinCc and rendered cells less sensitive to MinCD overproduction, the FtsZ C-terminal mutants were still sensitive to MinCn and did not form minicells, suggesting that they are still affected by MinC and that there is at least one more MinC interaction site on FtsZ [30]. This site was found to be located in a α-helix (H10) that is located at the interface of FtsZ subunits in polymerized FtsZ [31]. A mutation found in this loop rendered FtsZ less sensitive to MinC/MinD overexpression, increased polar divisions, and abolished the FtsZ-MinC interaction found in *in vitro* assays [31].

The interaction between *B. subtilis* FtsZ and MinC has not been characterized, but is assumed to be similar to that described for *E. coli* based on the homology between the systems. Here, we describe a similar approach as used by Shen and Lutkenhaus [30], [31] to generate MinC insensitive mutants of *B. subtilis* FtsZ, with the important difference that we used overproduction of full length MinC for mutant selection. In total, we found three FtsZ mutants that confer resistance to MinCD overproduction, and we characterized the effect of MinC on the mutants *in vitro*.

## Results

### Identification of ftsZ mutants that are insensitive to MinC

The aim of this study was to learn more about the interaction of FtsZ and its inhibitor MinC. To do this, we developed a screen for *ftsZ* mutants that confer insensitivity to MinC. Strain 1999 overexpresses GFP-MinC and MinD when xylose is added to the growth medium. GFP-MinC is fully functional and GFP-MinC/MinD overexpression causes filamentous growth and eventually lysis in liquid medium, and abrogates growth on plates [35]. Chromosomal DNA from earlier identified *ftsZ* mutants as well as DNA from a mutagenized plasmid library [36] was transformed to strain 1999 and the transformants were plated in the presence of xylose. After transformation, these strains contain a mutated, full length copy of *ftsZ* transcribed from its natural promoter, and a second copy of *ftsZ*, which is not expressed as it lacks its start codon, promoter and ribosome-binding site [see 36]. After control experiments, in which the retention of GFP-MinC/MinD overexpression was confirmed by checking for the presence of GFP-MinC, and correct integration of the *ftsZ* mutants in the chromosome was confirmed by a backcross experiment, three *ftsZ* mutants remained that allowed growth in the presence of elevated MinC/MinD. These mutants were the *ftsZ4*, *ftsZ8* and *ftsZ38* mutations that were already identified in the published screen [36]. The use of the randomly mutagenized plasmid library did not lead to the identification of new *ftsZ* mutations. In the published study, the *ftsZ4*, *ftsZ8* and *ftsZ38* mutants were identified as impaired in sporulation, either through a reduction of expression of the sporulation specific SpoIIQ protein (*ftsZ4*) or further impaired sporulation in a *minD* (*ftsZ38*) or *minCD* (*ftsZ8*) background [36]. Notably, this study already hinted at a reduced *minCD* sensitivity for these mutants as all showed an increased frequency in minicell formation in a wild type background (minicelling frequencies of ∼10%; ∼5% and <1% for *ftsZ4*, *ftsZ38* and *ftsZ8*, respectively). However, two other mutations that also caused minicell formation in a wild-type background, *ftsZ3* and *ftsZ24* [36], did not confer resistance to GFP-MinC/MinD overexpression.

### MinC insensitivity of ftsZ mutants

**Figure 1:**
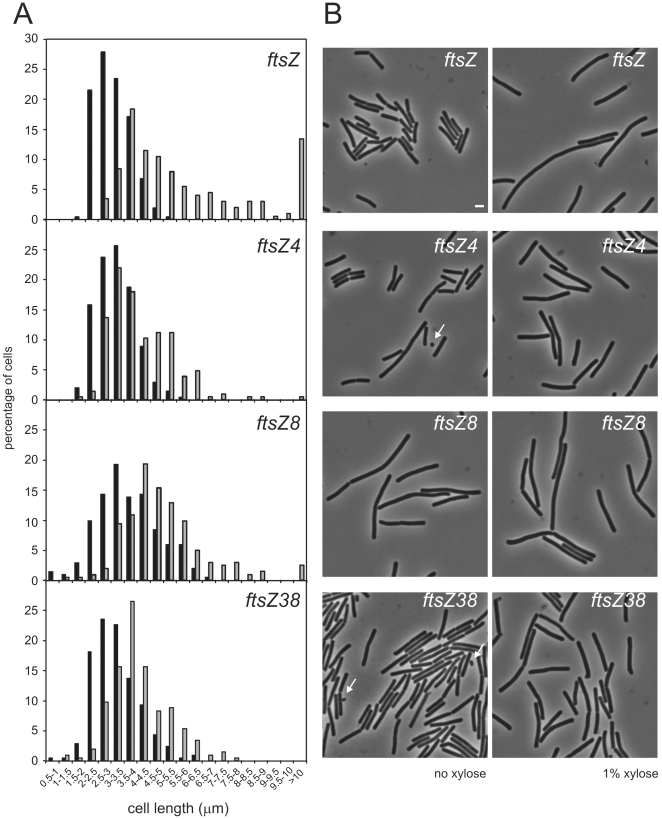
GFP-MinC/MinD overexpression leads to filamentation in cells expressing wild type FtsZ, but not in cells expressing FtsZ mutants. (A) Length distributions are shown of strains expressing either wild type or mutant *ftsZ*, after dilution of exponentially growing cells into fresh growth medium with (grey bars) or without (black bars) 1% Xylose to induce GFP-MinC/MinD overexpression. Cells were labelled with FM4.64 after 3.5 hours, incubated for another 30 minutes, fixed and processed for microscopy. Cell lengths were determined and grouped in length classes of 0.5 µm (more than 200 cells for each condition).(B) Phase contrast images of the cells used for the length distributions depicted in (A). Scale bar (same for all): 2 µm. Arrows indicate minicells.

To characterize the MinC-sensitivity of the *ftsZ* mutants in more detail we compared filamentation upon GFP-MinC/D overexpression of a strain expressing wild type *ftsZ* (in the same genetic background) with the strains expressing the *ftsZ* mutants. All strains were grown on liquid medium with or without induction of GFP-MinC/D and the length of >200 individual cells was measured (Fig. 1, Fig. S1). As was expected, the cells expressing wild type *ftsZ* became longer following GFP-MinC/MinD expression, with a large spread in the length distribution and a significant percentage of cells (>13%) forming filaments with lengths of 10 µm or longer (Fig. 1A, Fig. S1). In the absence of inducer, minicells could be readily observed in the *ftsZ4* and *ftsZ38* strains (Fig. 1B, arrows), as expected from the initially reported increase in minicelling frequency [36]. The strains expressing the *ftsZ* mutants were only slightly affected by the overexpression of GFP-MinC/MinD. In all cases the length distribution was shifted to the right, indicating that there is a minor effect of GFP-MinC/MinD expression on cell division. The increase in cell length was significant in all cases, but in contrast to cells expressing wildtype *ftsZ* the shape of the length distribution curve was not altered and, more important, long filaments were practically absent from the cultures (Fig. 1A, Fig. S1). Only *ftsZ8* did give rise to a few filaments of 10 µm or longer (<2.5%), but on the whole this variant of *ftsZ* had a wider length distribution, even in the absence of GFP-MinC/MinD expression. Using western blotting we established that the levels of FtsZ in all strains were similar (not shown), as was previously shown for these mutants in the *spoIIQ* reporter background [36]. This indicated that the refractory effect on GFP-MinC/MinD overexpression was not caused by elevated levels of FtsZ in the mutant strains. All mutants supported growth on plate at 30°C, 37°C and 43°C, indicating that the mutants are not temperature sensitive. Our results point to a reduced sensitivity to the cell division inhibitor MinC/MinD *in vivo* for the *ftsZ* mutants.

### Biochemical characterization of the FtsZ mutants

When mapped on the crystal structure of *B. subtilis* FtsZ [37], the FtsZ8 mutant, S219L, lies close to the active site for GTP hydrolysis. Mutations FtsZ4 (A285T) and FtsZ38 (L302P, with Q353R unresolved in the structure) lie on the outside of the FtsZ molecule. None of the mutations lie in the H10 helix or the conserved extreme C-terminus that were recently shown to interact with MinC in *E. coli* [30], [31]. We purified the mutant proteins to characterize their polymerization behavior and to test their MinC sensitivity *in vitro*.

**Figure 2:**
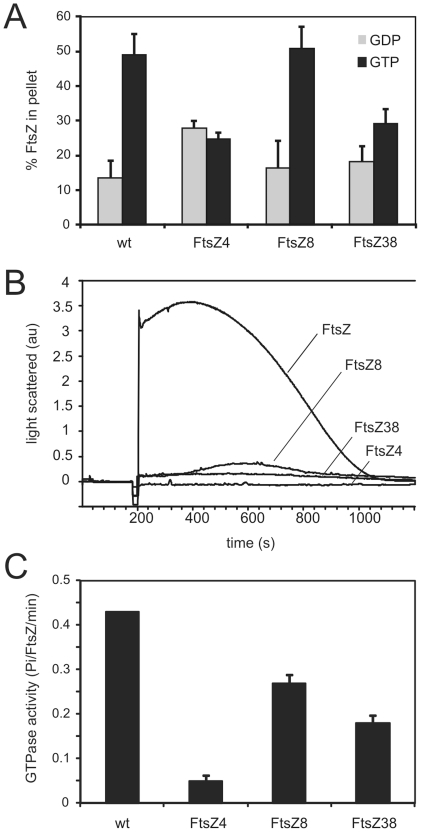
Polymerization and GTPase activity of the FtsZ mutants. (A) Sedimentation assay. Wild type and mutant FtsZ (10 µM) was incubated in 50 mM Hepes/NaOH; 50 mM KCl; 10 mM MgCl_2_; pH 7.5 polymerization buffer at 30°C. Polymerization was induced by addition of 1 mM GTP (or 1 mM GDP as control) and after 5 mins incubation at 30°C, polymers were recovered by ultracentrifugation. The amount of pelleted FtsZ was quantified as described in material and Methods. Mean + SD from three independent experiments are shown. (B) Light scattering. FtsZ (10 µM) was incubated in 50 mM Mes/NaOH; 50 mM KCl; 10 mM MgCl_2_; pH 6.5 polymerization buffer After 3 min. baseline recording, GTP was added to 1 mM. pH 6.5 polymerization buffer was used as this results in a more readily detectable light scattering signal [21], [38]. (C) GTP hydrolysis. Wild type and mutant FtsZ (10 µM) was incubated in 50 mM Hepes/NaOH; 50 mM KCl; 10 mM MgCl_2_; pH 7.5 polymerization buffer at 30°C. After addition of GTP to 1 mM, the release of phosphate was followed by a colorimetric assay and the GTPase activity was determined over 30 mins within the linear range of phosphate release. Mean + SD from three independent experiments are shown.

First we assayed FtsZ polymerization at pH 7.5 using sedimentation. FtsZ8 was found to behave similar to wild type FtsZ (Fig 2A). FtsZ4 was found to have a high background sedimentation in the presence of GDP, and the addition of GTP did not increase the amount of sedimented material, indicating that the sedimented material was either aggregated or consisted of polymers that form irrespective of the nucleotide added (no addition of nucleotide resulted in similar levels of sedimented material, not shown). FtsZ38 showed an increase in sedimented material upon the addition of GTP but again at relatively high background levels and with a total yield of sedimented material that was significantly lower than for the wild type protein. It has to be noted that in sedimentation assays DEAE-dextran, which promotes the formation of FtsZ filament bundles, is used to ensure efficient sedimentation [see also 21]. We also followed polymerization of the mutants using light scattering. The mutants showed either non-detectable (FtsZ4) or a very low increase (FtsZ8, FtsZ38) in the amount of light scattered upon the addition of GTP (Fig 2B.), even though FtsZ8 was found to polymerize efficiently by sedimentation (Fig. 2A). In order to detect any signal we resorted to performing the experiments at pH 6.5, which is known to increase scattering due to increased lateral association of FtsZ filaments (Fig. 2B)[21], [38]. The result indicates that the FtsZ mutants have a very low propensity for forming lateral bundles, which is missed by the sedimentation assay. The dynamics of FtsZ polymerization and GTP hydrolysis are tightly linked so we also determined the rate of GTP hydrolysis of the FtsZ mutants (Fig. 2C). We found that FtsZ8 and FtsZ38 both showed reduced GTP hydrolysis when compared to the wild type protein, whereas FtsZ4 seemed to have no GTP hydrolysis activity. The absence of GTP hydrolysis activity in FtsZ4 is in line with the observation that FtsZ4 does not display GTP dependent polymerization.

### Activity of the FtsZ mutants in the presence of MinC

**Figure 3:**
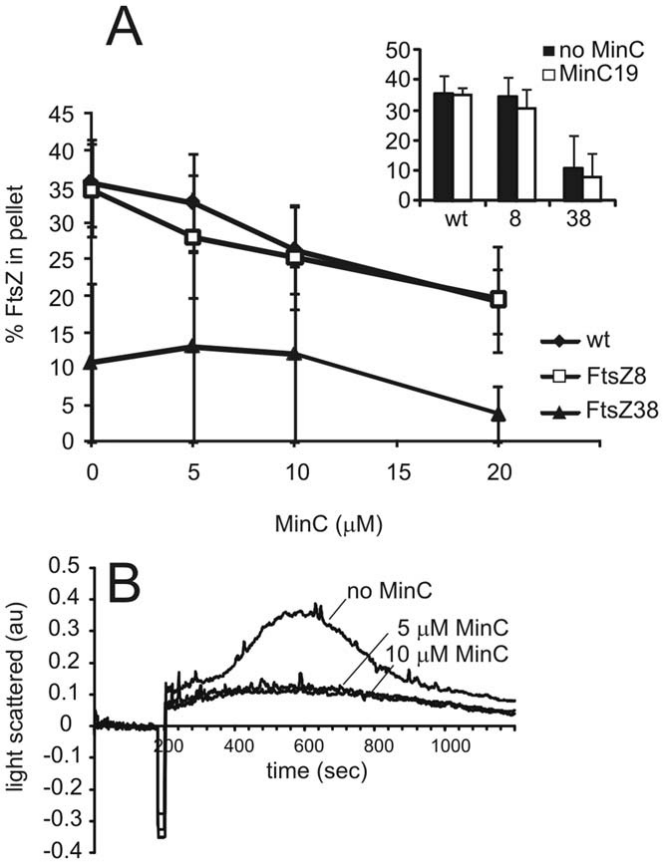
FtsZ8 and FtsZ38 are still sensitive to MinC. (A) Polymerization of FtsZ, FtsZ8 and FtsZ38 (10 µM) was determined in the presence of MinC at various concentrations (x-axis) by sedimentation as described for Fig. 2A. In this experiment, background sedimentation with GDP was subtracted to plot only GTP-dependent sedimentation of polymers. Inset: control experiment where polymerization was determined in the presence of the less active MinC19 mutant (20 µM). Mean ± SD from three independent experiments are shown. (B) FtsZ8 (10 µM) polymerization monitored by light scattering with MinC at concentrations indicated. The experiment was performed identical to the description in Fig 2B, the trace in the absence of MinC is duplicated – note the difference in scale.

As the FtsZ mutants are not affected by MinC overexpression *in vivo*, we wanted to test whether the polymerization activity of the mutants is also less sensitive to the presence of MinC *in vitro*. We determined the GTP-dependent polymerization activity of wild type FtsZ, FtsZ8 and FtsZ38 in the presence of MinC at various concentrations. FtsZ4 did not show detectable GTP-dependent polymerization (Fig. 2A) and so was excluded from this analysis. To our surprise, we found that FtsZ8 polymerization is inhibited by MinC to a similar extent as wild type FtsZ (Fig. 3A). Although the light scattering of FtsZ8 signal was very low compared to wild type (Fig. 2B), an inhibitory effect of MinC could also be detected by light scattering (Fig. 3B). FtsZ38 does not show very efficient polymerization, however it seems less affected by the presence of MinC. Polymerization is not reduced at a 1∶1 ratio of FtsZ38 to MinC (at 10 µM), but does decrease when MinC is present in two-fold excess. As FtsZ38 polymerization was not detected with light scattering (Fig. 2B) we could not confirm the result using this technique. As a control, we performed polymerization assays of the FtZ mutants in the presence of two-fold excess of the less active MinC19 mutant [21]. As expected, polymerization was hardly affected by the MinC19 mutant (Fig. 3, inset). We also analyzed the GTP hydrolysis activity of the FtsZ mutants in the presence of MinC. This was, like wild type GTP hydrolysis activity, unchanged (data not shown)[21].

### Electron microscopy of the FtsZ mutants

**Figure 4:**
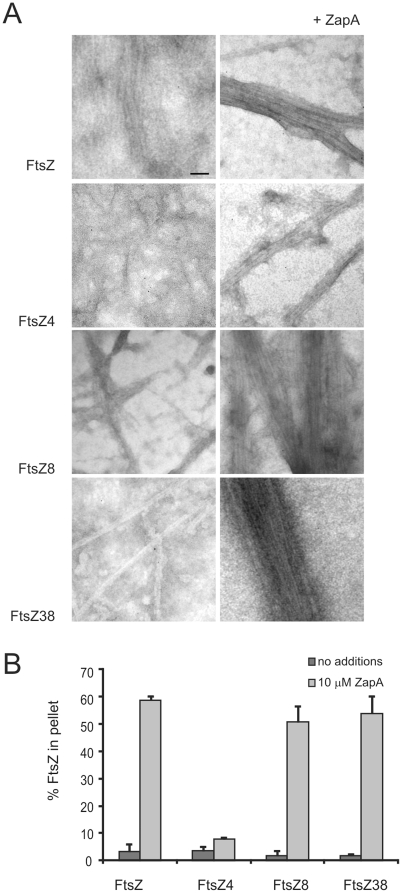
ZapA enhances bundle formation and polymerization of the FtsZ mutants. (A) FtsZ and the FtsZ mutants (10 µM) were polymerized as described in the text, in the absence (left column) or presence (right column) of ZapA (10 µM). 5 minutes after GTP addition, samples were processed for electron microscopy. Scale bar (same for all) 100 nm. (B) Polymerization of wild type FtsZ and the FtsZ mutants as determined by sedimentation in the absence or presence of ZapA (10 µM). Mean + SD from three independent experiments are shown.

The sedimentation and light scattering analysis of polymerization did not reveal why our FtsZ mutants are more refractive to excess MinC in vivo. It could be that the insensitivity to MinC is not caused by a direct change in the MinC-FtsZ interaction, but by a stronger interaction of FtsZ with accessory proteins that stimulate FtsZ polymerization. This was tested for ZapA, which is known to bundle FtsZ [39] and counteract the effects of MinC on FtsZ [21]. As we did not observe extensive polymerization at pH 7.5 we switched to a pH 6.5 buffer which is more conducive to polymerization. At pH 6.5, polymerization was observed for wild type FtsZ, FtsZ8 and FtsZ38, but not for FtsZ4 (Fig. 4A). Adding ZapA, however, restored polymerization capability to FtsZ4. The same polymerization mixtures were analyzed by sedimentation. In all cases, the bundling of FtsZ filaments by ZapA, allowed the recovery of polymerized FtsZ above background levels without having to add DEAE-dextran to the sedimentation mixture as described before [21]. It is interesting to note that FtsZ38, which sedimented less efficiently than wt FtsZ in experiments without ZapA (but with DEAE-dextran), sediments with similar efficiency in the presence of ZapA.

### The FtsZ mutants do not depend on the presence of other cell division proteins

As ZapA restored full polymerization capability to FtsZ38 and partial capability to FtsZ4, we speculated that the FtsZ mutants might require the presence of ZapA *in vivo*. If so, introducing the FtsZ mutations in a *zapA* deletion background would confer synthetic lethality. A similar result could be expected if the FtsZ mutants would require the presence of other positive regulators of FtsZ, like SepF [34] or FtsA [40] that are required for efficient Z-ring assembly. We introduced the *ftsZ* mutations, as well as wild type *ftsZ* in the same genetic background, into strains containing deletions of *zapA*, *sepF*, or *ftsA*. To our surprise, none of these transformations resulted in synthetic lethality. We then determined the growth rates of these strains on two types of media to see if the *ftsZ* mutants conferred any growth disadvantage to these strains. No essential changes in doubling time were observed between strains carrying wild type or mutant *ftsZ* when grown on LB (Table 1). When grown on minimal medium, it appears that in a *ftsA* background, the *ftsZ8* and *ftsZ38* mutants grow slightly slower than the wild type. These results indicate that the MinC resistant phenotype of the *ftsZ* mutants is not caused by the stronger dependence on the presence of known positive regulators of FtsZ.

**Table 1:** Growth rates.

*ftsZ*			*zapA::tet*		*ftsA::erm*		*sepF::pMutin4*	
	LB	MM	LB	MM	LB	MM	LB	MM
*wild type*	20±2	27±3	22±2	30±4	30±4	39±2	21±2	42±2
*ftsZ4*	20±2	28±4	20±1	26±1	27±6	37±2	20±1	37±2
*ftsZ8*	21±3	30±2	21±1	31±3	31±5	45±3	20±2	36±1
*ftsZ38*	23±2	29±4	22±2	33±4	29±8	45±4	21±2	43±4

Doubling times, and standard deviations (in minutes) as determined for three independent growth curves for the *ftsZ* mutants in cell division attenuated strains grown on Luria Bertani broth (LB) or minimal medium (MM).

## Discussion

In this study we have identified, isolated and characterized mutations that render *B. subtilis* FtsZ resistant to the overexpression of MinC-MinD. The aim of this mutant search was to identify mutations in FtsZ that would abolish the interaction between FtsZ and MinC, in order to obtain information about the contact interface between the two proteins. Our strategy was quite similar to that employed by Shen and Lutkenhaus, who have used overexpression of the C- and N-terminal domains of *E. coli* MinC to identify two sites on FtsZ that interact with MinC: an interaction site located on the FtsZ C-terminal tail that also binds FtsA and ZipA, and an interaction site located at the interface of FtsZ subunits in the polymer [30], [31]. The *in vivo* resistance observed for the *E. coli* FtsZ mutations was mirrored by a decreased sensitivity to MinC in *in vitro* polymerization assays, and a failure to bind MinC in pull down assays [30], [31]. Our mutations did not confer such a clear-cut *in vitro* phenotype and are therefore less readily explained.

We used a previously described strategy to generate random mutations in *ftsZ* [36]. Subsequently, we selected for *ftsZ* mutations that confer resistance to overproduction of GFP-MinC/MinD. As overproduction of GFP-MinC/MinD is lethal [35] and MinC can directly inhibit FtsZ polymerization [21], the rationale behind the selection was that isolated FtsZ mutants should no longer be sensitive to MinC *in vitro*. The screen is designed such that control of *ftsZ* expression is through the wildtype promoter and the *ftsZ* mutant is the only version of *ftsZ* on the chromosome. This prevents the selection of strains that have become resistant to elevated levels of MinC by overproducing FtsZ. We confirmed that FtsZ cellular levels were the same for all strains as was previously reported [36]. Notably, although the original screen was aimed at selecting *ftsZ* mutations that are affected in sporulation, we did not identify any new mutants in *ftsZ.* In fact, although 5 mutants in the previous screen displayed a minicelling phenotype, indicative of resistance to MinC/MinD, only three of those also conferred resistance to GFP-MinC/MinD overproduction. We confirmed the Min-resistance phenotype by overproducing GFP-MinC/MinD during growth and following cell length distribution. In a wild type background, overproduction of GFP-MinC/MinD led to a block in cell division and therefore rapid formation of elongated filaments. All the mutants showed mild increases in length upon GFP-MinC/MinD overproduction, but long filaments were absent, indicating that these strains could still successfully complete division.

Since we have recently described *in vitro* assays for FtsZ polymerization inibition by MinC [21], we isolated the FtsZ mutant proteins and performed similar experiments. Notably, all mutants appear defective in polymerization as compared to wild type FtsZ. First, mutant FtsZ4 did not appear to polymerize in a GTP-dependent manner, either when assayed by sedimentation or light scattering. In fact, this mutant was only found to form polymers, detectable by electron microscopy, when ZapA was added. FtsZ8 and FtsZ38 did show GTP-dependent polymer sedimentation, to different levels, but when studied by light scattering it was obvious that both mutants only give very small scattering signals compared to wild type FtsZ. As polymers can be observed by EM we attribute this decrease in scattering signal to a reduction in the formation of lateral associations between the polymer filaments. It has to be noted that, although the net polymerization determined by sedimentation for FtsZ8 was the same as wild type, this experiment is done in the presence of DEAE-dextran which enhances bundle formation of FtsZ polymers. This suggests that similar amounts of FtsZ8 and wild type FtsZ are involved in polymer formation, but that the increased lateral associations of wild type FtsZ are responsible for the increased light scattering signal. To our surprise, the polymerization of FtsZ8 as detected by sedimentation was affected to a similar extent as wild type FtsZ by the presence of MinC. It did seem that, although the total amount of protein polymerized was significantly lower, FtsZ38 is more refractive to MinC as the addition of MinC to FtsZ38 at stoichiometric amounts did not have an effect. Unfortunately, we did not have another assay to quantitatively determine the effect of MinC on the FtsZ mutants, as MinC does not affect FtsZ GTP hydrolysis activity (which we confirmed for the mutants). We have tried, but not been able, to establish a direct assay for FtsZ-MinC interaction by incubation of purified FtsZ and MinC-strep using various conditions and cross-linking, followed by purification of MinC-strep with streptactin beads. Notably, we have also never been able to recover MinC from the pellet of FtsZ-MinC polymerization reactions, as is possible with the *E. coli* proteins [7]. It is possible that the affinity between MinC and FtsZ is weaker in *B. subtilis* than in *E. coli*, but in the absence of a reliable assay we cannot speculate on a dimished interaction between MinC and the FtsZ mutants.

The FtsZ mutants do not map in regions that are known to be involved in interactions between FtsZ and MinC, or FtsZ and itself. FtsZ4, A285T, maps in a loop region connecting H10 with S9 [37]. It could be that the presence of a hydroxyl group near H10 changes the ability of H10 to participate in an FtsZ-FtsZ interaction. Mutations in H10 in *E. coli* FtsZ are responsible for loss of interaction with MinC [31]. Although the mutation does not affect cell division or growth rate *in vivo*, it had the most drastic effect *in vitro*, as polymerization could only be detected after addition of ZapA and GTP hydrolysis was blocked. FtsZ8, S219L, maps in a loop between H8 and S7. This mutation leads to a wider distribution of cell lengths but not to altered growth rates. However, FtsZ8 responds similar to wild type FtsZ in MinC inhibition assays, and the major difference with wild type FtsZ seems the loss of lateral associations as determined by light scattering. FtsZ38 has two mutations, L302P and Q353R. Q353R is part of the disordered C-terminus of FtsZ, but not of the extreme C-terminal tail which is the interaction platform for many proteins like EzrA, SepF, FtsA, and, in *E. coli*, MinC and ZipA [see 30]. L302P is located in a loop between S9 and S10. Interestingly, L302 lines the binding pocket for 3-methoxybenzamide (3-MBA) and its derivative compound PC190723 which have shown to be potent inhibitors of cell division and *in vitro* GTP hydrolysis activity [37]. Binding of these compounds has been proposed to alter the orientation between domains of FtsZ and thus inhibit FtsZ activity. It could be that the L302P mutation also affects this orientation - the mutation affects both polymerization and GTP hydrolysis activity.

It is likely that the mutations we have identified are not conferring resistance to MinC through a loss of interaction with MinC but rather through an alteration of polymerization properties that renders the FtsZ polymers insensitive to the action of MinC. This is similar to the initial Min resistant FtsZ mutations identified by Bi and Lutkenhaus [26], [27]. It could be that the overexpression of full-length MinC that we employed is too toxic to screen for mutations that have solely lost the ability to interact with MinC, especially if, like in *E. coli*, the interaction is mediated by two sites in the protein that can partially compensate each other [30], [31]. We find it interesting to note that all our mutants have retained the ability to polymerize and bundle in the presence of ZapA, but do not seem to form lateral associations when analysed on their own by light scattering. Since MinC disrupts lateral interactions of FtsZ [20] it could be that the reduced bundling capacity of the mutants precludes the action of MinC. However, one would expect that this reduced ability to bundle would be reflected by an increased dependency on other FtsZ stabilizing proteins such as ZapA, FtsA or SepF, which we did not observe. On the whole it seems that the formation of the FtsZ-ring in *B. subtilis* is very robust and that the bacterium can easily cope with either mutations in FtsZ or the absence of accessory proteins.

## Materials and Methods

### Strains, plasmids, growth conditions

All strains and plasmids are listed in Table 2. *B. subtilis* cells were made competent for transformation with DNA either by the method of Kunst and Rapoport [41], or by the method of Anagnostopoulos and Spizizen [42] as modified by Jenkinson [43]. Correct integration of DNA into the chromosome after transformation of *B. subtilis* was checked by PCR amplification. DNA manipulations and *E. coli* transformations were carried out using standard methods [44]. Liquid medium used for growing *B. subtilis* was either Penassay Broth (PAB, Oxoid Antibiotic medium no. 3), Luria Bertani broth (LB), or minimal medium (MM, Spizizen salts supplemented with 0.5% w/v glucose; 0.02% w/v casein hydrolysate and 0.02% L-tryptophan); solid medium was LB with 1.5% w/v agar, with antibiotics added as required. Chloramphenicol was used at 5 µg/ml, spectinomycin at 50 µg/ml, erythromycin at 0.5 µg/ml, lincomycin at 12.5 µg/ml, and kanamycin at 5 µg/ml. Liquid medium used for *E. coli* was Luria Bertani broth (LB), and solid medium was LB with 1.5% w/v agar, with antibiotics and glucose (0.5% w/v) added as required. Ampicillin was used at 100 µg/ml, spectinomycin at 50 µg/ml.

**Table 2:** Strains and plasmids.

Strain/plasmid	Relevant characteristics	Construction/reference
***B. subtilis***		
168	*trpC2*	laboratory collection
1999	*trpC2 Ω amyE::spc P_xyl_-gfp-minC minD*	[35]
FG356	*zapA-yshBΔ::tet Ω amyE::P_xyl_-zapA cat Ω thrC::P_spac_-yshB erm*	[39]
BFA2863	*sepF:(pMUTIN Pspac-ylmG erm)*	BFA project, [47]
YK206	*CRK6000 ftsA::erm Pspac-ftsZ*	[34]
3970	*trpC2 Ω ftsZ::pSG1928 ftsZ cat*	3975 → 168 (Cm)
3971	*trpC2 Ω ftsZ::pSG1928 ftsZ4 (A285T) cat*	3976 → 168 (Cm)
3972	*trpC2 Ω ftsZ::pSG1928 ftsZ8 (S219L) cat*	3977 → 168 (Cm)
3974	*trpC2 Ω ftsZ::pSG1928 ftsZ38 (L302P; Q353R) cat*	3979 → 168 (Cm)
3975	*1999 Ω ftsZ::pSG1928 ftsZ cat*	pSG1928 [36] → 1999 (Cm)
3976	*1999 Ω ftsZ::pSG1928 ftsZ4 (A285T) cat*	1358 [36] → 1999 (Cm)
3977	*1999 Ω ftsZ::pSG1928 ftsZ8 (S219L) cat*	1365 [36] → 1999 (Cm)
3979	*1999 Ω ftsZ::pSG1928 ftsZ38 (L302P; Q353R) cat*	1368 [36] → 1999 (Cm)
3990	*trpC2 zapA-yshBΔ::tet Ω ftsZ::pSG1928 ftsZ cat*	FG356 → 3970 (Tc)
3991	*trpC2 zapA-yshBΔ::tet Ω ftsZ::pSG1928 ftsZ4 (A285T) cat*	FG356 → 3971 (Tc)
3992	*trpC2 zapA-yshBΔ::tet Ω ftsZ::pSG1928 ftsZ8 (S219L) cat*	FG356 → 3972 (Tc)
3994	*trpC2 zapA-yshBΔ::tet Ω ftsZ::pSG1928 ftsZ38 (L302P; Q353R) cat*	FG356 → 3974 (Tc)
4010	*trpC2 sepF:(pMUTIN Pspac-ylmG erm) Ω ftsZ::pSG1928 ftsZ cat*	BFA2863 → 3970 (erm)
4011	*trpC2 sepF:(pMUTIN Pspac-ylmG erm) Ω ftsZ::pSG1928 ftsZ4 (A285T) cat*	BFA2863 → 3971 (erm)
4012	*trpC2 sepF:(pMUTIN Pspac-ylmG erm) Ω ftsZ::pSG1928 ftsZ8 (S219L) cat*	BFA2863 → 3972 (erm)
4014	*trpC2 sepF:(pMUTIN Pspac-ylmG erm) Ω ftsZ::pSG1928 ftsZ38 (L302P; Q353R) cat*	BFA2863 → 3974 (erm)
4020	*trpC2 ftsA::erm Ω ftsZ::pSG1928 ftsZ cat*	YK206 → 3970 (erm)
4021	*trpC2 ftsA::erm Ω ftsZ::pSG1928 ftsZ4 (A285T) cat*	YK206 → 3971 (erm)
4022	*trpC2 ftsA::erm Ω ftsZ::pSG1928 ftsZ8 (S219L) cat*	YK206 → 3972 (erm)
4024	*trpC2 ftsA::erm Ω ftsZ::pSG1928 ftsZ38 (L302P; Q353R) cat*	YK206 → 3974 (erm)
***E. coli***		
BL21 (DE3)		[48]
DH5α	F-*endA1 hsdR17 supE44 thi-1* λ*-recA1 gyrA96 relA1* Δ(*lacZYA-argF*)*U169 Φ80 dlacZ* Δ*M15*	GIBCO-BRL
**Plasmids**		
pCXZ	*bla P_tac_ -ftsZ_BS_*	[45]
pBS58	*spc ftsQAZ_EC_*	[45]
pSG5332	*bla P_tac_-ftsZ4*	this work
pSG5333	*bla P_tac_-ftsZ8*	this work
pSG5349	*bla P_tac_-ftsZ38*	this work
pDJ15	*bla tetR PtetA-minC-strep-tagII*	[21]
pDJ16	*bla tetR PtetA-minC19-strep-tagII*	[21]
pDJ26	*bla lacI^Q^ P_T7_-his_8_-zapA*	[21]

### Plasmid construction

Plasmids pSG5332, pSG5333 and pSG5349 were constructed to overexpress FtsZ4, FtsZ8 and FtsZ38 respectively and are derivatives of pCXZ [45]. The mutant *ftsZ* genes were amplified by PCR from chromosomal DNA from strains 3976, 3977 and 3979 using primers T3 (5′- AATTAACCCTCACTAAAGGG) and DJS161 (5′- CTGCAGGTCGACGGATCCCGGGAATAGATAGATAGTCATTCGGC). DJS161 binds upstream of *ftsZ* and introduces a *Sal*I site, and T3 binds downstream of *ftsZ* and the multiple cloning site of the pSG1928 mutator plasmid that was inserted in the chromosome. The resulting PCR products were digested with *Sal*I and *Eco*RI, making use of a *Eco*RI site in the multiple cloning site of pSG1928, and cloned into *Sal*I-*Eco*RI digested pCXZ.

All constructs were checked by DNA sequencing.

### Cell length determination

Exponentially growing cells in PAB medium were diluted back into fresh PAB medium with or without 1% xylose to inducce overexpression of GFP-MinC/MinD. After 3.5 hours of growth, cells were labelled with FM4.64 (0.5 µg/ml; Molecular Probes), grown for another 30 mins, fixed, and processed for microscopy. Microscopy was performed as described [46], using an Olympus BX-60 fluorescence microscope equipped with a UPLANFI 100x/1.3 oil objective and a Photometrics Coolsnap-*fx* CCD camera. Cell lengths were determined using the ObjectImage software package (N. Vischer, University of Amsterdam, http://simon.bio.uva.nl/object-image.html).

### Protein purification

FtsZ, the FtsZ mutants, MinC, MinC19 and ZapA were all purified essentially as described [21]. FtsZ4 and FtsZ38 were found to aggregate upon freezing/thawing as well as during prolonged storage at 4°C in storage buffer (20 mM Tris/HCl; 1 mM EDTA; 2.5 mM MgAc; 10% v/v glycerol pH 7.5). Adding KCl to 200 mM and storage at 4°C prevented aggregation.

### FtsZ polymerization and GTP hydrolysis

Sedimentation assays, light scattering, GTP hydrolysis assays and electron microscopy were essentially performed as described [21]. FtsZ4 and FtsZ38 preparations were subjected to an initial centrifugation step (15 min, 154,000 *g*, 4°C) to remove any potential aggregated material, and the protein concentration of the supernatant was determined, before use in subsequent assays. Protein concentrations and buffer compositions are mentioned in the text.

## Supporting Information

Figure S1 A box-plot showing the length distribution data displayed in Figure 1A. Strains expressing either wild type or mutant ftsZ were grown in rich medium to exponential phase and diluted into fresh growth medium with or without 1% Xylose to induce GFP-MinC/MinD overexpression. Cells were labelled with FM4.64 after 3.5 hours, incubated for another 30 minutes, fixed and processed for microscopy. Cell lengths were determined for 200 or more cells per population (total number between brackets per population on y-axis). A boxplot was generated using the SPSS Statistics 17 software package. Each box is delimited by the first and third quartiles, the line crossing the box is the median. The whiskers correspond to 1.5 times the interquartile range (IQR). Minor outliers, between 1.5 and 3 times the IQR outside the central box, are denoted as circles, major outliers, 3 times or more the IQR are denoted as asterixes. Length distributions of each strain grown in the absence or presence of inducer were analyzed for similarity using a Mann-Whitney test in SPSS, and in each case the difference between the length distribution found with induction was significantly different from the length distribution without induction (p<0.0005 for all four strains).
(0.97 MB TIF)
