# Supplementary figures and images for: Characterization of ftsZ Mutations that Render Bacillus subtilis Resistant to MinC
Source: PLoS One. 2010 Aug 11;5(8):e12048. doi: 10.1371/journal.pone.0012048 (PMC2920321; doi:10.1371/journal.pone.0012048)

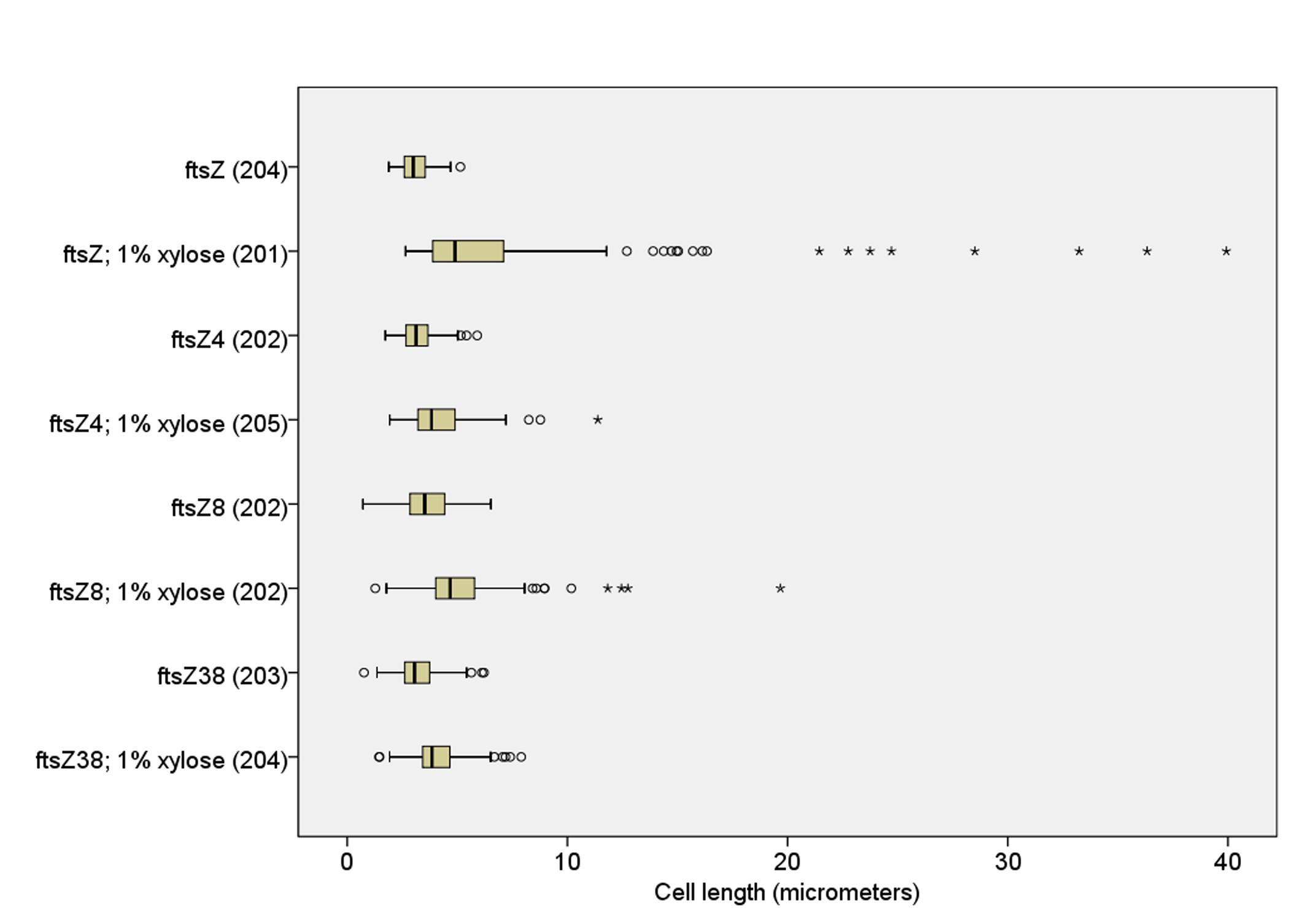

Supplement: Figure S1 — A box-plot showing the length distribution data displayed in Figure 1A. Strains expressing either wild type or mutant ftsZ were grown in rich medium to exponential phase and diluted into fresh growth medium with or without 1% Xylose to induce GFP-MinC/MinD overexpression. Cells were labelled with FM4.64 after 3.5 hours, incubated for another 30 minutes, fixed and processed for microscopy. Cell lengths were determined for 200 or more cells per population (total number between brackets per population on y-axis). A boxplot was generated using the SPSS Statistics 17 software package. Each box is delimited by the first and third quartiles, the line crossing the box is the median. The whiskers correspond to 1.5 times the interquartile range (IQR). Minor outliers, between 1.5 and 3 times the IQR outside the central box, are denoted as circles, major outliers, 3 times or more the IQR are denoted as asterixes. Length distributions of each strain grown in the absence or presence of inducer were analyzed for similarity using a Mann-Whitney test in SPSS, and in each case the difference between the length distribution found with induction was significantly different from the length distribution without induction (p<0.0005 for all four strains). (0.97 MB TIF) [file pone.0012048.s001.tif]
